# Elevated Eosinophil Counts in Acute Exacerbations of Bronchiectasis: Unveiling a Distinct Clinical Phenotype

**DOI:** 10.1007/s00408-023-00668-w

**Published:** 2024-01-16

**Authors:** Weixin Chen, Siyi Ran, Chenchang Li, Zhixin Li, Nili Wei, Jing Li, Naijian Li

**Affiliations:** 1 Department of Chinese and Western Medicine in Clinical Medicine, The Clinical School of Chinese and Western Medicine of Guangzhou Medical University, Guangzhou, People’s Republic of China; 2 State Key Laboratory of Respiratory Disease, Department of Allergy and Clinical Immunology, National Center for Respiratory Medicine, Guangzhou Institute of Respiratory Health, The First Affiliated Hospital of Guangzhou Medical University, Guangzhou, People’s Republic of China; 3 Johns Hopkins Asthma & Allergy Center, Johns Hopkins University School of Medicine, Baltimore, MD USA

**Keywords:** Eosinophils, Bronchiectasis, Clinical features, Phenotype

## Abstract

**Background:**

Non-cystic fibrosis bronchiectasis is a chronic respiratory disease characterized by bronchial dilation. However, the significance of elevated eosinophil counts in acute exacerbations of bronchiectasis remains unclear.

**Methods:**

This retrospective case-control study included 169 hospitalized patients with acute exacerbations of non-cystic fibrosis bronchiectasis. Based on blood eosinophil levels, patients were categorized into eosinophilic and non-eosinophilic bronchiectasis groups. Various clinical variables, including lung function, comorbidities and clinical features were collected for analysis. The study aimed to examine the differences between these groups and their clinical phenotypes.

**Results:**

Eosinophilic bronchiectasis (EB) was present in approximately 22% of all hospitalized patients with bronchiectasis, and it was more prevalent among male smokers (*P < *0.01). EB exhibited greater severity of bronchiectasis, including worse airway obstruction, higher scores in the E-FACED (FACED combined with exacerbations) and bronchiectasis severity index (BSI), a high glucocorticoids medication possession ratio, and increased hospitalization cost (*P* < 0.05 or *P* < 0.01). Furthermore, we observed a significant positive correlation between blood eosinophil count and both sputum eosinophils (r = 0.49, *P* < 0.01) and serum total immunoglobulin E levels (r = 0.21, *P* < 0.05). Additional analysis revealed that patients with EB had a higher frequency of shortness of breath (*P < *0.05), were more likely to have comorbid sinusitis (*P < *0.01), and exhibited a greater number of lung segments affected by bronchiectasis (*P* < 0.01).

**Conclusions:**

These findings suggest that EB presents a distinct pattern of bronchiectasis features, confirming the notion that it is a specific phenotype.

## Background

Non-cystic fibrosis (Non-CF) bronchiectasis is a chronic respiratory disease characterized by bronchial dilation, which can lead to chronic cough, sputum production, and recurrent respiratory infections [1]. Previous studies have identified the significant role of neutrophils in the pathophysiology of bronchiectasis [2]. Elevated levels of neutrophils in the airways can cause chronic inflammation, tissue damage, and progressive decline in lung function. Neutrophilic inflammation also serves as a marker for disease severity and a predictor of exacerbations [3, 4]. Therapies targeting neutrophils, such as inhaled antibiotics and azithromycin, may effectively reduce airway inflammation and improve outcomes in some patients with non-cystic fibrosis bronchiectasis [5].

However, elevated eosinophil counts have been observed in the sputum and blood of certain patients with Non-CF bronchiectasis, suggesting the presence of a specific subtype of the disease [6, 7]. This type of bronchiectasis, characterized by high levels of eosinophils, is known as eosinophilic bronchiectasis (EB). Currently, there are limited studies on EB, and the value of an elevated eosinophil count as a biomarker for clinical, diagnostic, or prognostic assessment of bronchial dilation remains unclear. Bronchiectasis is a complex and heterogeneous disease with various clinical phenotypes and endotypes. The identification of potentially treatable traits could aid in personalizing treatment and improving patient outcomes [2]. This retrospective study aimed to examine the clinical phenotype of eosinophilic bronchiectasis and compare it to non-eosinophilic bronchiectasis, highlighting their similarities and differences.

## Methods

### Study Design and Patients

This study utilized a retrospective case-control study design. From January 2021 to January 2023, a total of 215 patients with acute exacerbations of non-cystic fibrosis bronchiectasis were admitted to the First Affiliated Hospital of Guangzhou Medical University, National Center for Respiratory Medicine, Guangzhou, P.R. China. The diagnosis of bronchiectasis was based on the European Respiratory Society guidelines for the management of adult bronchiectasis [1]. The study included patients who met the following criteria for study entry: (1) Aged between 18 and 75 years; (2) Non-CF bronchiectasis diagnosed for more than one year using high-resolution computerized tomography (HRCT); (3) Availability of searchable medical records within the most recent year; (4) Hospitalization for the treatment of acute bronchiectasis exacerbation. Exclusion criteria were as follows: (1) History of cancer, asthma, active allergic bronchopulmonary aspergillosis (ABPA), or other clinically significant lung diseases apart from bronchiectasis; (2) Presence of severe heart or respiratory failure; (3) Missing necessary clinical and laboratory test data. This study received approval from the institutional review board of Guangzhou Medical University Hospital (No: 2022-055). Informed patient consent was waived due to the retrospective nature of the study.

**Fig. 1:**
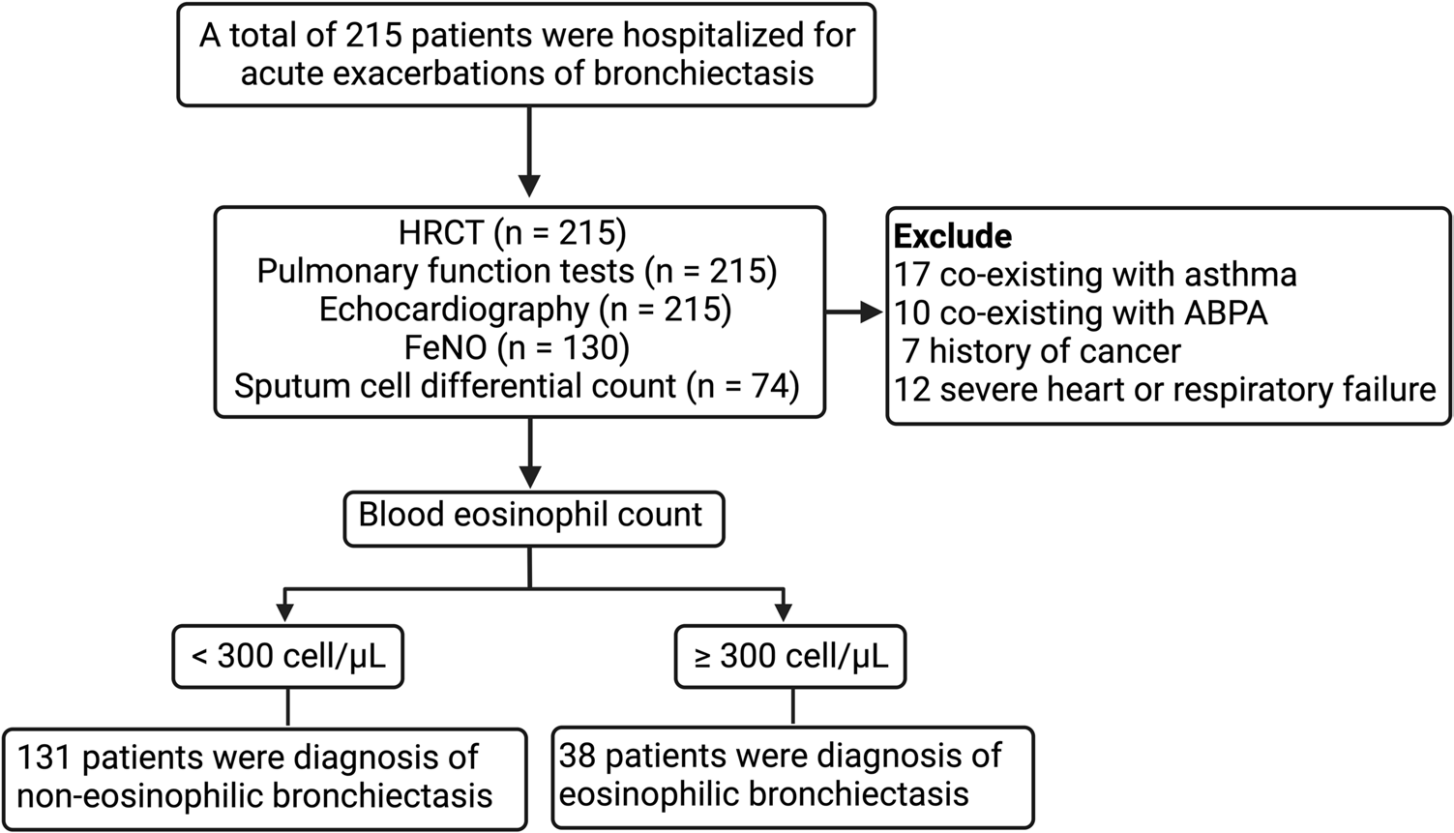
Flowchart of cohort included in the study. A total of 215 patients with non-cystic fibrosis bronchiectasis were initially enrolled, but 46 patients with specific conditions including asthma, ABPA, cancer history, and severe heart or respiratory failure were subsequently excluded

This comprehensive flowchart provides a clear overview of the patient selection process and diagnostic outcomes in the study (Fig. 1). Initially, a total of 215 patients were enrolled, all of whom were hospitalized for acute exacerbations of bronchiectasis. Subsequently, 46 patients with specific conditions, including asthma, ABPA, a history of cancer, and severe heart or respiratory failure, were excluded from the study. The diagnostic process involved various assessments for the remaining 169 patients. High-resolution computed tomography, pulmonary function tests, and echocardiography were conducted for all 215 patients. Fractional exhaled nitric oxide (FeNO) measurements were available for 130 patients, while sputum cell differential counts were obtained for 74 patients. The final diagnostic outcomes revealed that 131 patients were diagnosed with non-eosinophilic bronchiectasis, while 38 patients were diagnosed with eosinophilic bronchiectasis.

### Study Definitions and Variables

The patients who met the inclusion criteria were categorized into two or three groups based on their eosinophil levels, whether below or above a specific threshold. In this study, EB was defined as blood eosinophilia ≥ 300 cells/µl, while non-eosinophilic bronchiectasis (NEB) was defined as blood eosinophilia  < 300 cells/µl [6, 7]. Sputum eosinophilia was defined as eosinophils  > 3% of total cells [8]. To explore the impact of different cut-off values of blood eosinophils in patients with bronchiectasis, we also employed pre-established cutoffs widely acknowledged in the COPD literature: less than 100 cells/µl, 100 to 299 cells/µl, and greater than 300 cells/µl [9].

General clinical information was collected from all study patients according to standard procedures by the First Affiliated Hospital of Guangzhou Medical University. This information included anthropometry, smoking history, medication history, lung function, symptoms, radiologic features, the number of exacerbations and hospitalizations for exacerbations, hospitalization cost, and inflammatory blood cell counts. Furthermore, two comprehensive measures assessing the severity of bronchiectasis were derived from the aforementioned variables: E-FACED (FACED combined with exacerbations) and the Bronchiectasis Severity Index (BSI) [10].

### Statistical Methods

Depending on the distribution of the variables, quantitative data were tabulated using either the mean (standard deviation [SD]) or the median (interquartile range). The differences between the two clusters of patients were evaluated using the chi-square test. In the case of comparing three groups, appropriate statistical tests such as ANOVA were utilized. Associations between clinical and biological variables were examined using Pearson’s correlation coefficient. Statistical analysis was performed using SPSS version 27 (IBM SPSS, Armonk, NY, USA), and a corrected *p*-value of < 0.05 was considered statistically significant.

## Results

### Characteristics of the Study Population

Initially, 215 patients with Non-CF bronchiectasis and available blood eosinophil counts were enrolled. Subsequently, 17 patients with asthma, 10 with ABPA, 7 with a history of cancer, and 12 with severe heart or respiratory failure were excluded. As a result, a total of 169 patients were included in the study. Among these bronchiectasis patients, 64 (37.9%) were males and 105 (62.1%) were females. The mean age of the included patients was 55.95 ± 14.66 years, and the mean disease duration was 12.8 years. Regarding baseline lung function, the patients with bronchiectasis had a mean FEV1% of 79.43 ± 27.50, a mean FVC% of 89.27 ± 22.18, and a mean FEV1/FVC% of 72.16 ± 14.46. Bronchiectasis is highly prevalent in middle-aged and elderly women. Compared to the normal population, bronchiectasis patients exhibited reduced levels of FEV1% and FEV1/FVC%. The median percentage of blood eosinophil count was 2.3% (1.2–3.9), with a median absolute number of 140 (90–265) cells/µl.

### Relationship Between Blood Eosinophil Count and Disease Phenotype

Table 1 presents the distribution of blood eosinophil counts in the following groups: 131 (77.5%) with < 300 cells/µl and 38 (22.5%) with ≥ 300 cells/µl. A larger proportion of males represented patients with EB, and their smoking index was significantly higher compared to patients with non-eosinophilic bronchiectasis (NEB) (*P* < 0.01). When comparing the EB group with the NEB group, the former exhibited a greater overall severity of bronchiectasis. The EB group showed lower lung function (*P* < 0.01), a higher glucocorticoid medication possession ratio (*P* < 0.05), worse Bronchiectasis Severity Index (BSI) and E-FACED scores (*P* < 0.001). Furthermore, the EB group also had a higher rate of chronic rhinosinusitis (*P* < 0.05), serum total IgE level (*P* < 0.01), and high-sensitivity C-reactive protein (HS-CRP) level (*P* < 0.05). Comparing EB to NEB, we observed no significant difference in blood neutrophil counts. However, the proportion of neutrophils was notably higher in NEB compared to EB (*P* < 0.05). There were no significant differences in age, BMI, disease duration, Pseudomonas aeruginosa (PA) infection rate, and fractional exhaled nitric oxide (FeNO) level between the patients with EB and the NEB group. Hospitalization cost were also significantly increased in the EB group compared to the NEB group. Interestingly, the EB group had a lower nontuberculous mycobacteria (NTM) infection rate (*P* < 0.01). Overall, there was a correlation between the severity of bronchiectasis and an increase in blood eosinophil count levels.

**Table 1:** Characteristics of bronchiectasis patients stratified by blood eosinophil count

Characteristics	Blood eosinophil counts		*P* value
	< 300 Cells/µl	≥ 300 Cells/µl	
No. of subjects	131	38	
Age, years	55.5 ± 14.8	57.5 ± 14.2	0.467
Disease during years	10 (6–18)	9 (5–20)	0.364
Gender, M, n (%)	42 (32.1)	22 (57.9)	0.004
BMI, kg/m^2^	21.1 ± 3.9	22.1 ± 3.9	0.126
Smoking history, n (%)	20 (15.3)	14 (36.8)	0.024
Smoking index (pack-yr)	12.3 ± 10.4	20.4 ± 14.0	0.025
FEV_1_ (L)	2.02 ± 0.82	1.76 ± 0.80	0.086
FEV_1_% predicted	82.52 ± 26.39	68.81 ± 28.95	0.006
FVC (L)	2.70 ± 0.86	2.60 ± 0.88	0.548
FVC% predicted	91.32 ± 20.96	82.20 ± 24.99	0.025
FEV_1_/FVC%	73.78 ± 13.87	66.58 ± 15.27	0.007
MMEF 75/25(L/s)	1.76 ± 1.16	1.22 ± 0.84	0.008
BSI	8.4 ± 2.6	10.4 ± 3.1	< 0.001
E-FACED	3.2 ± 1.5	4.7 ± 1.8	< 0.001
Chronic rhinosinusitis, n (%)	36 (27.5)	20 (52.6)	0.004
PA infection, n (%)	10 (7.6)	4 (10.5)	0.958
NTM infection, n (%)	16 (12.2)	0 (0)	0.024
Glucocorticoids MPR, n (%)	31 (23.7)	16 (42.1)	0.025
FeNO (ppd)	19.4 ± 11.3	18.9 ± 15.9	0.912
NE (cells/µl)	420 (300–580)	430 (360–680)	0.225
NE (%)	65.1 (56.3–72.1)	58.8 (50.8–67.9)	0.032
HS-CPR (mg/L)	2.2 (1.1–9.4)	5.5 (2.8–23.1)	0.028
Total IgE (kU/L)	44 (16–85)	53 (23–162)	0.043
Hospitalization cost (RMB)	15,360 ± 5763	19,056 ± 11,811	0.032

Data are shown as means ± SD or median (interquartile range). Differences in continuous variables were evaluated using Wilcoxon test or chi-square test.

*BMI* Body mass index, *BSI* Bronchiectasis severity index, *E-FACED* Forced expiratory volume in 1 S; age; chronic colonization by Pseudomonas aeruginosa; radiological extension (number of pulmonary lobes affected); dyspnea, *FeNO* Fractional exhaled nitric oxide, *HS-CPR* Hypersensitive C-reactive protein, *MPR* Medication possession ratio, *MMEF* Maximal mid-expiratory flow curve, *NE* Neutrophils, *NTM* Nontuberculous mycobacteria, *PA* Pseudomonas aeruginosa, *RMB* Renminbi

In the above analysis, we used a blood eosinophil count of 300 cells/µl as a criterion to analyze the clinical characteristics of patients with EB or NEB. To investigate the impact of different blood eosinophil cut-off values on the bronchiectasis phenotype, we referred to accepted cut-off values from other studies on eosinophil-induced bronchiectasis: < 100 cells/µl, 100–299 cells/µl, and ≥ 300 cells/µl [6]. These different cut-off values were applied to examine the clinical characteristics of patients with varying blood eosinophil levels (Table 2). Our study revealed minimal differences between the results of the < 100 cells/µl group and the 100–299 cells/µl group in terms of clinical features. However, patients with EB still exhibited significant differences compared to patients with other eosinophil levels. These findings suggest that eosinophilic bronchiectasis has a distinct phenotype.

**Table 2:** Characteristics of bronchiectasis patients stratified by blood eosinophil count in three cohorts

Characteristics			Blood Eosinophil Counts			*P* value
			< 100 Cells/µl	100–299 Cells/µl	≥ 300 Cells/µl	
No. of subjects			55	76	38	
Age, years			56.0 ± 15.0	55.2 ± 14.7	57.5 ± 14.2	NS
Disease during years			9 (5–19)	10 (7–17)	9 (5–20)	NS
Gender, M, n (%)			17 (30.9)	25 (32.9)	22 (57.9)	< 0.01^bc^
BMI, kg/m^2^			20.8 ± 3.6	21.3 ± 3.6	22.1 ± 3.9	NS
Smoking history, n (%)			9 (16.4)	11 (14.5)	14 (36.8)	< 0.05^bc^
Smoking index (pack-yr)			13.5 ± 11.5	11.3 ± 12.2	20.4 ± 14.0	< 0.05^bc^
FEV_1_ (L)			1.97 ± 0.81	2.06 ± 0.83	1.76 ± 0.80	NS
FEV_1_% predicted			83.50 ± 25.98	81.80 ± 26.83	68.81 ± 28.95	< 0.05^bc^
FVC (L)			2.59 ± 0.84	2.77 ± 0.87	2.60 ± 0.88	NS
FVC% predicted			90.73 ± 20.93	91.75 ± 21.10	82.20 ± 24.99	< 0.05^c^
FEV_1_/FVC%			75.00 ± 12.48	72.89 ± 14.80	66.58 ± 15.27	< 0.05^bc^
MMEF 75/25(L/s)			1.74 ± 1.12	1.78 ± 1.20	1.22 ± 0.84	< 0.05^bc^
BSI			8.6 ± 2.5	8.2 ± 2.7	10.4 ± 3.1	< 0.01^bc^
E-FACED			3.3 ± 1.6	3.2 ± 1.5	4.7 ± 1.8	< 0.01^bc^
Chronic rhinosinusitis, n (%)			14 (25.4)	20 (28.9)	20 (52.6)	< 0.05^bc^
PA infection, n (%)			5 (9.1)	5 (6.6)	4 (10.5)	NS
NTM infection, n (%)			6 (10.9)	10 (13.2)	0 (0)	< 0.05^b^
Glucocorticoids MPR, n (%)			15 (29.1)	16 (21.1)	16 (42.1)	< 0.05^b^
FeNO (ppd)			18.1 ± 11.2	19.4 ± 11.9	18.9 ± 15.9	NS
NE (cells/µl)			460 (310–670)	410 (280–530)	430 (360–680)	NS
NE (%)			70.8 (61.6–76.1)	61. 9(53.4–68.8)	58.8 (50.8–67.9)	< 0.05^bc^
HS-CPR (mg/L)			1.7 (0.8–9.2)	4.7 (1.5–13.0)	5.5 (2.8–23.1)	< 0.05^b^
Total IgE (kU/L)			46 (15–77)	36 (19–106)	53 (23–162)	< 0.05^bc^
Hospitalization cost (RMB)			15,780 ± 5953	14,404 ± 4776	19,056 ± 11,811	< 0.01^b^

Data are shown as means ± SD or median (interquartile range). Differences in continuous variables were evaluated using ANOVA, and *p* values were corrected using the Bonferroni method.

^a^*P* < 0.05 for equality between the EOS < 100 Cell/µL group and EOS 100–299 Cell/µL. ^b^*P* < 0.05 for equality between the EOS < 100 Cell/µL group and EOS ≥ 300 Cell/µL. ^c^*P* < 0.05 for equality between the EOS 100–299 Cell/µL group and EOS ≥ 300 Cell/µL.

*BMI* Body mass index, *BSI* Bronchiectasis severity index, *E-FACED* Forced expiratory volume in 1 S; age; chronic colonization by Pseudomonas aeruginosa; radiological extension (number of pulmonary lobes affected); dyspnea, *FeNO* Fractional exhaled nitric oxide, *NE* Neutrophils, *HS-CPR* Hypersensitive C-reactive protein, *MPR* Medication possession ratio, *MMEF* Maximal mid-expiratory flow curve, *NTM* Nontuberculous mycobacteria, *NS* No significant difference, *PA* Pseudomonas aeruginosa, *RMB* Renminbi

### Correlation Between Blood Eosinophil Counts with Sputum Eosinophil and Total IgE

**Fig. 2:**
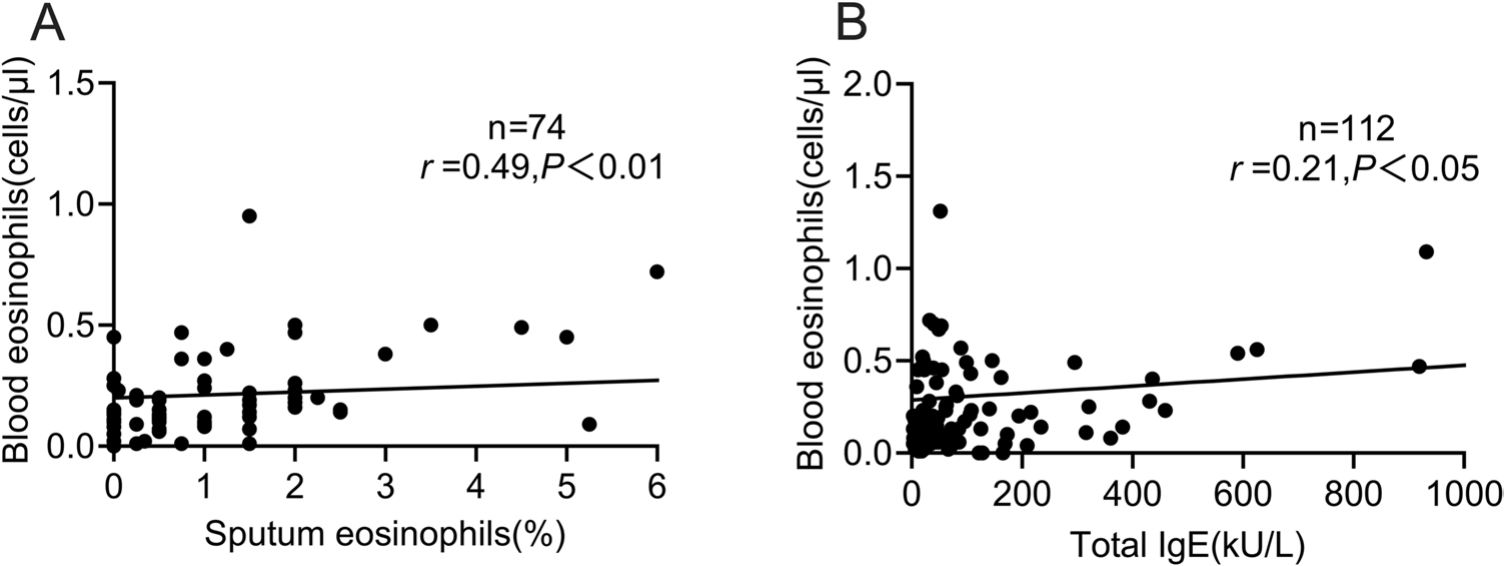
Correlation between blood eosinophil counts with sputum eosinophil and total IgE. **A** Correlation between blood and sputum eosinophil counts (r = 0.49; *P* < 0.01). **B** Correlation between blood eosinophil counts and total immunoglobulin E levels (r = 0.21; *P* < 0.05)

To investigate the relationship between blood eosinophil counts and airway inflammation, we analyzed the blood eosinophil counts, sputum eosinophil counts, and serum total IgE levels. Among the 169 bronchiectasis patients included in the study, 74 underwent induced sputum examinations, and 112 underwent detection of serum total IgE levels. The results revealed a significant positive correlation between blood eosinophil count and sputum eosinophils (r = 0.49, *P* < 0.01, Fig. 2A). Similarly, there was a significant positive correlation between blood eosinophil counts and serum total IgE levels (r = 0.21, *P* < 0.05, Fig. 2B).

### Relationship Between Blood Eosinophil Counts and Clinical Characteristics in Bronchiectasis

There were no significant differences between patients with EB and those with NEB in terms of clinical symptoms such as cough, coughing sputum, chest pain, and hemoptysis. However, patients with EB exhibited a higher prevalence of dyspnea (*P* < 0.05). Similarly, there were no significant differences between the two groups in pulmonary signs (Table 3).

**Table 3:** Clinical features of patients grouped by blood eosinophil count

Clinical features	Blood eosinophil counts		*P* value
	< 300 Cell/µL	≥ 300 Cell/µL	
Symptom			
Cough, n (%)	115 (87.8)	37 (97.4)	0.155
Coughing sputum, n (%)	89 (67.9)	30 (78.9)	0.191
Dyspnea, n (%)	22 (16.8)	17 (34.2)	0.020
Chest pain, n (%)	5 (4)	0 (0)	0.589
Hemoptysis, n (%)	32 (24.6)	8 (21.1)	0.390
Signs			
Dry rales, n (%)	9 (7)	4 (10.5)	0.690
Wet rales, n (%)	50 (38.2)	18 (47.4)	0.111
Localization of Bronchiectasis			
Upper localization bronchiectasis, n (%)	75 (52.3)	32 (84.2)	< 0.01
Middle/lingular localization bronchiectasis, n (%)	89 (67.9)	32 (84.2)	0.051
Basal localization bronchiectasis, n (%)	70 (53.4)	38 (100)	< 0.01
Bilateral bronchiectasis, n (%)	70 (53.4)	33 (86.8)	< 0.01
Widespread bronchiectasis, n (%)	32 (24.4)	27 (71.1)	< 0.01
Number of lung segments with bronchiectasis	2 (1–5)	5 (3–5)	< 0.01

Data are shown as means ± SD or median (interquartile range). Differences in continuous variables were evaluated using Wilcoxon test or chi-square test

Next, we investigated whether there was a correlation between eosinophilic inflammation and the localization of bronchiectasis in patients with a blood eosinophil count of ≥ 300 cells/µl. All 169 bronchiectasis patients included in the study underwent chest CT imaging, and the diagnostic imaging reports were used for analysis (Table 3). Among the cases of EB, basal bronchiectasis was present in 100% of the cases, while widespread bronchiectasis was observed in 71.1% of the cases. Further analysis revealed that 84.2% of the cases showed upper or middle/lingular localization, and 86.8% of the cases exhibited bilateral localization. The number of lung segments with bronchiectasis was significantly higher in EB compared to NEB. An increase in blood eosinophils ≥ 300 cells/µl appears to be associated with more extensive and multiple preferential localization of bronchiectasis (*P* < 0.05 or *P* < 0.01). This finding suggests a potential new radiological phenotype that may require a dedicated therapeutic strategy in the future.

## Discussion

Bronchiectasis is a complex and heterogeneous disease with various clinical phenotypes and endotypes. The aim of this study was to examine the clinical phenotype of EB and compare it with NEB. The study design employed a retrospective case-control approach, analyzing data from 169 patients hospitalized for acute exacerbations of non-cystic fibrosis bronchiectasis. Patients were categorized into EB and NEB groups based on blood eosinophil levels, with a threshold of ≥ 300 cells/µl for EB and  < 300 cells/µl for NEB. Our findings demonstrate that EB represents a distinct phenotype within the spectrum of bronchiectasis, characterized by elevated blood eosinophil counts and unique clinical characteristics.

In bronchiectasis, studies have shown varying prevalence of eosinophilia, with approximately 20.4% of non-asthmatic or allergic bronchopulmonary aspergillosis-related bronchiectasis patients exhibiting peripheral blood eosinophilia [6]. In this study, the prevalence of EB in bronchiectasis patients is 22.5%, which is consistent with findings from previous limited studies. High blood eosinophil count in adults is independently associated with male sex, current smoking, positive skin prick test, COPD, and asthma [11]. Consistent with the aforementioned studies, the EB patients in this study exhibited similar characteristics, including male smokers, elevated levels of total IgE, and reduced lung function.

The association between peripheral eosinophil count and exacerbations in bronchiectasis, along with the optimal cut-off point that accurately reflects this relationship, remains a topic of debate [12]. In a study conducted by Kwok et al. [13], focusing on Chinese patients, the role of blood eosinophil counts in hospitalizations due to bronchiectasis exacerbations was investigated. The study findings suggest that an eosinophil count of 250 cells/µl is the threshold that identifies a higher risk of hospitalization for bronchiectasis. In the current study, we employed pre-established cut-offs that are widely acknowledged in the literature on COPD and asthma [9, 14], and these cutoffs are also consistent with those reported in a European Multicohort Study [6]. However, future research should explore different cut-off values to validate and refine the definition of EB.

One of the key findings of this study is the association between blood eosinophil count and the severity of bronchiectasis. Patients with EB exhibited greater overall disease severity, as evidenced by lower lung function, higher glucocorticoid medication usage, worse scores on bronchiectasis severity indices (BSI and E-FACED), and increased hospitalization cost. This observation suggests that blood eosinophil count could serve as a potential biomarker for disease severity in bronchiectasis, aiding in risk stratification and treatment decision-making.

Several studies have demonstrated a correlation between severe eosinophilic inflammation and disease progression in respiratory conditions like asthma and COPD [15–18]. This association could be attributed to the involvement of eosinophils in airway remodeling. Eosinophils can secrete matrix metalloproteinases, promoting fibroblast activation and differentiation into myofibroblasts, both of which contribute to airway remodeling [19]. Additionally, eosinophils are stimulated by pathogens to release eosinophil extracellular traps (EET), accompanied by the release of exosomes and elastase, which further impact airway remodeling and extracellular matrix changes [20]. However, various studies have demonstrated the bactericidal and antiviral properties of eosinophils, both during stable phases and exacerbations [21]. This finding may provide an explanation for the lower incidence of NTM infection observed in the EB group.

It is important to note that our results did not reveal an association between EB and the Pseudomonas aeruginosa infection rate. Firstly, the size and specific characteristics of our study cohort may differ from those in other studies, contributing to variations in observed associations [22, 23]. Additionally, inhaled corticosteroids (ICS) use is associated with an increased risk of Pseudomonas aeruginosa (PA) infection [24, 25]. Furthermore, in this study, the diagnosis of Pseudomonas aeruginosa infection relied on positive sputum bacterial culture results. Unlike the high-throughput sequencing method, the positive rate of sputum culture was markedly lower. Consequently, the Pseudomonas aeruginosa infection rate in this study was also significantly reduced. This discrepancy constitutes one of the reasons why our findings do not suggest an association between eosinophilic disease and the Pseudomonas aeruginosa infection rate.

The infiltration of eosinophils significantly influences airway remodeling and, consequently, lung function levels, resulting in eosinophilic bronchiectasis displaying notably lower lung function compared to other bronchiectasis subtypes. The correlation between blood eosinophil counts and sputum eosinophil counts, as well as serum total IgE levels, further supports the role of eosinophilic inflammation in EB. The positive correlation between these parameters indicates a close relationship between blood and airway eosinophilia, emphasizing the involvement of eosinophils in the pathogenesis of EB. The identification of EB as a distinct phenotype within bronchiectasis has important implications for personalized treatment strategies. Current therapies targeting neutrophils, such as inhaled antibiotics and liposomal ciprofloxacin, may not be effective for EB patients [26, 27]. Instead, therapies that specifically target eosinophilic inflammation, such as corticosteroids or monoclonal antibodies against interleukin-5, could be explored as potential treatment options for EB [28, 29]. A single-center observational cross-sectional study, including 249 patients, reported bronchiectasis predominantly characterized by type 2 immune response (peripheral blood eosinophil count ≥ 300/µl or FeNO ≥ 25 ppb) [30]. In the case series of severe bronchiectasis, treatment with mepolizumab or benralizumab significantly reduced exacerbation rates for up to 2 years. These findings suggest the importance of T2-high endotype-targeted biological treatments in bronchiectasis patients.

We acknowledge several limitations in our study. Firstly, it was a retrospective study conducted at a single center, and the sample size was small, which may introduce selection bias. Secondly, we did not assess blood or sputum inflammatory markers to evaluate the impact of type 2 inflammation on bronchodilation. Third, this study did not investigate the potential relationship between the phenotype of eosinophilic bronchiectasis and the microbiota. Despite these limitations, our study provides valuable insights into the topic, and future research should aim to address these shortcomings.

## Conclusions

The retrospective case-control study provided insights into the clinical characteristics and phenotypes of eosinophilic and non-eosinophilic bronchiectasis. Elevated eosinophil levels were found in a subset of patients, suggesting the existence of a distinct subtype of the disease. The findings emphasize the importance of further research to understand the role of eosinophils as potential biomarkers and personalized treatment targets in bronchiectasis.

## Data Availability

The raw data supporting the conclusions of this article will be made available by the authors, without undue reservation.

## Abbreviations

ABPA: Active allergic bronchopulmonary aspergillosis
BMI: Body mass index
BSI: Bronchiectasis severity index
COPD: Chronic obstructive pulmonary disease
EB: Eosinophilic bronchiectasis
EET: Eosinophil extracellular traps
E-FACED: Forced expiratory volume in 1 S; age; chronic colonization by Pseudomonas aeruginosa; radiological extension (number of pulmonary lobes affected); dyspnea
FeNO: Fractional exhaled nitric oxide
FEV1: Forced expiratory volume in the first second
FVC: Forced vital capacity
HS-CRP: High-sensitivity C-reactive protein
MMEF: Maximal mid-expiratory flow curve
MPR: Medication possession ratio
NE: Neutrophils
NEB: Non-eosinophilic bronchiectasis
NTM: Non-eosinophilic bronchiectasis
NTM: Nontuberculous mycobacteria
PA: Pseudomonas aeruginosa
RMB: Renminbi
